# CTCF interacts with the lytic HSV-1 genome to promote viral transcription

**DOI:** 10.1038/srep39861

**Published:** 2017-01-03

**Authors:** Fengchao Lang, Xin Li, Olga Vladimirova, Benxia Hu, Guijun Chen, Yu Xiao, Vikrant Singh, Danfeng Lu, Lihong Li, Hongbo Han, J. M. A. S. P. Wickramasinghe, Sheryl T. Smith, Chunfu Zheng, Qihan Li, Paul M. Lieberman, Nigel W. Fraser, Jumin Zhou

**Affiliations:** 1 Key Laboratory of Animal Models and Human Disease Mechanisms of the Chinese Academy of Sciences & Yunnan Province, Kunming Institute of Zoology, Kunming, Kunming 650223, China; 2 Kunming College of Life Science, University of Chinese Academy of Sciences, Kunming, Beijing 100101, China; 3 Gene Expression and Regulation Program, The Wistar Institute, Philadelphia, PA 19104, USA; 4 Biology & Chemistry Engineering College, Panzhihua University, Panzhihua, Sichuan 617000, China; 5 Department of Biology, Arcadia University, Glenside, PA 19038, USA; 6 Institutes of Biology and Medical Sciences, Soochow University, Suzhou, 215123, China; 7 Department of Microbiology, Immunology and Infectious Diseases, University of Calgary, Calgary, AB T2N 4N1, Canada; 8 Department of Viral Immunology, Institute of Medical Biology, Chinese Academy of Medicine Science, Peking Union Medical College, Kunming, Kunming 650118, China; 9 Department of Microbiology, Perelman School of Medicine, University of Pennsylvania, Philadelphia, PA 19104, USA

## Abstract

CTCF is an essential chromatin regulator implicated in important nuclear processes including in nuclear organization and transcription. Herpes Simplex Virus-1 (HSV-1) is a ubiquitous human pathogen, which enters productive infection in human epithelial and many other cell types. CTCF is known to bind several sites in the HSV-1 genome during latency and reactivation, but its function has not been defined. Here, we report that CTCF interacts extensively with the HSV-1 DNA during lytic infection by ChIP-seq, and its knockdown results in the reduction of viral transcription, viral genome copy number and virus yield. CTCF knockdown led to increased H3K9me3 and H3K27me3, and a reduction of RNA pol II occupancy on viral genes. Importantly, ChIP-seq analysis revealed that there is a higher level of CTD Ser2P modified RNA Pol II near CTCF peaks relative to the Ser5P form in the viral genome. Consistent with this, CTCF knockdown reduced the Ser2P but increased Ser5P modified forms of RNA Pol II on viral genes. These results suggest that CTCF promotes HSV-1 lytic transcription by facilitating the elongation of RNA Pol II and preventing silenced chromatin on the viral genome.

The CCCTC binding factor, CTCF, is an essential epigenetic regulator with multiple nuclear functions. It was first identified as a transcription factor interacting with the c-*myc* promoter, but its defining property is chromatin insulation i.e. preventing the activity of a transcriptional enhancer from acting beyond CTCF binding sites to activate transcription^1^,^2^, or to prevent the spread of silenced chromatin, or heterochromatin. Indeed, genome-wide studies revealed that CTCF binding often occurs near the boundaries of silenced chromatin mark H3K27me3 and heterochromatin mark H3K9me3^3^,^4^,^5^. Genome-wide analyses also suggest that CTCF may directly regulate gene transcription, as its binding sites have been seen at gene promoters and enhancers^6^. Molecular studies also implicated a role of CTCF in regulating RNA Pol II recruitment, elongation and pausing^7^,^8^. Today, CTCF is believed to play a global role in organizing the genome into elaborate topological loops and domains to facilitate long distance interactions and keep regulatory activities in check^9^,^10^. ChIP-seq studies have revealed a CTCF DNA binding consensus sequence of about 20 bp^11^, but a recent report suggests that regions containing CTCF binding sites likely interact with additional proteins to provide directionality, thus specificity of looping^12^. However, how CTCF exerts diverse functions is not well understood.

CTCF has been reported to interact with or regulate several DNA viruses including Herpes Simplex Virus (HSV)-1, Epstein–Barr virus (EBV), Kaposi’s sarcoma-associated herpesvirus (KSHV), Human cytomegalovirus (HCMV) and adenovirus^13^,^14^,^15^,^16^,^17^,^18^,^19^. CTCF interacts with the latent HSV-1 genome and may control its latency by protecting an area of active chromatin around the LAT promoter from silencing^20^,^21^,^22^. In EBV and KSHV, CTCF is reported to play a role in regulating viral gene expression during latent infection and maintaining epigenetic states, including chromatin loop structures, while in HCMV, CTCF binding sites directly regulate CMV gene transcription^13^,^14^,^17^,^23^,^24^.

During HSV-1 lytic infection, host transcription machinery and accessory factors are essential for making viral immediate early, early and late transcripts^25^,^26^,^27^. Cellular RNA polymerase II, especially the CTD ser2P modified form, is essential for viral gene transcription^28^,^29^,^30^. At the chromatin level, HSV-1 uses host chromatin assembly factors, host histones and histone variants to assemble chromatin^31^,^32^,^33^. Host histone acetyltransferases are also needed to create a transcription permissive environment for viral transcription^34^,^35^, as are demethylases^36^. Before and during HSV-1 enters replication, viral immediate early (IE) or early (E) protein interact with cellular proteins to form nuclear structures referred to as replication compartments^37^,^38^,^39^. As a key cellular epigenetic regulator, CTCF has been reported to interact with several sites with HSV-1 genome during latency and reactivation^21^,^22^,^40^. However, whether CTCF plays a role in HSV-1 lytic infection is not known. Here we present evidence that CTCF is recruited to the HSV-1 genome at multiple binding sites that differ from previously reported sites and localizes to a substructure within the viral replication compartments. Knockdown of CTCF led to a reduction of viral transcription, replication, and virus yield. We demonstrated that CTCF supports HSV-1 transcription by preventing the spreading of repressive histone marks and promoting the recruitment of RNA Pol II, especially the elongating form to viral genes.

## Results

### CTCF is recruited to HSV-1 replication compartments

**Figure 1:**
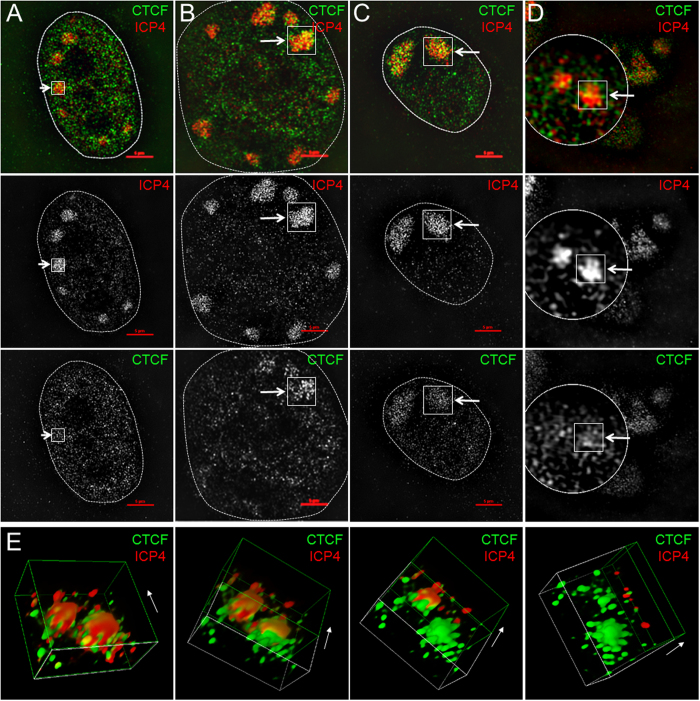
CTCF is recruited to HSV-1 replication compartments. Human primary fibroblast BJ cells were infected with HSV-1 17+ at an MOI of 5 for 6 hours and were stained with antibody against CTCF (green) and antibody against ICP4 (red). Imaging was done with a Nikon N-SIM laser microscopy at the same magnification. **(A)** The ICP4 signal labeled the early stage of HSV-1 replication compartments and showed very little sign of CTCF recruitment. **(B)** In intermediate sized HSV-1 replication compartments, significant amount of CTCF is detected (arrow). **(C)** In more mature HSV-1 replication compartments, there is strong recruitment of CTCF (arrow). **(D)** Higher magnifications of two viral replication compartment showing colocalization between ICP4 and CTCF. **(E)** 3-D reconstruction of the region described in (**D**). ICP4 staining is gradually removed digitally. Arrow indicates the direction of peeling.

To explore the spatial relationship between CTCF and HSV-1 genome, we performed super resolution immunofluorescence microscopy (Nikon N-SIM) imaging in human primary fibroblast BJ cells at 6 hours post infection (hpi, Fig. 1). HSV-1 replication compartments were visualized with antibody to ICP4 or ICP8 (Fig. 1, Supplementary Figures S1 and S2), which is known to bind the viral genome during lytic infection^41^,^42^. At early stage of viral genome synthesis, replication compartments appeared small and showed no obvious visual colocalization with CTCF (Fig. 1A). As the viral replication compartment grew larger, colocalization between CTCF enriched foci and ICP4 labeled replication compartment became apparent (Fig. 1B,C). In larger, more developed HSV-1 replication compartments, a significant amount of CTCF recruitment into the replication compartment was seen (Fig. 1C). At higher magnification, and in 3-D reconstruction images CTCF could be seen to form an organized mesh-like structure in and around the viral replication foci, while ICP4 aggregated into a more solid structure (Fig. 1D,E). In the 3-D reconstructed images, it appears that CTCF wraps around a core structure formed by ICP4, as ICP4 staining is gradually removed digitally (Fig. 1E). Since ICP4 presumably labels viral DNA that is committed to transcription, we also labeled HSV-1 replication compartments with ICP8^42^, a single-stranded DNA binding protein marking the replicating HSV-1 DNA, and found colocalization of CTCF with the ICP8 labeled viral replication compartments (Supplementary Figure S1). In contrast to traditional fluorescent microscopy (Supplementary Figure S2), we noted that CTCF signals did not colocalize precisely with either ICP4 or ICP8 within the replication compartments under super resolution microscopy (Fig. 1E and Supplementary Figure S1). These data suggest that CTCF might localize to distinct substructure from ICP4 or ICP8 within the HSV-1 replication compartments.

### The zinc finger domain of CTCF is necessary for its recruitment to HSV-1 replication compartments

**Figure 2:**
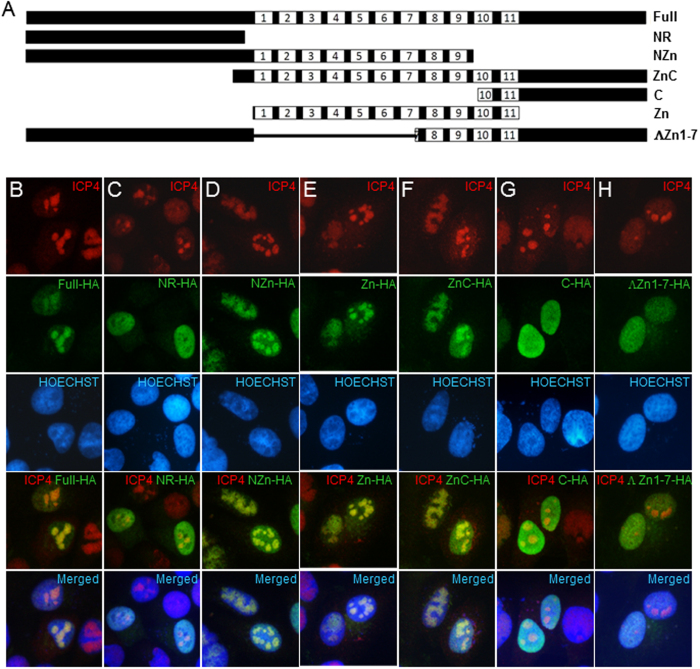
The zinc finger region is necessary for efficient recruitment of CTCF. **(A)** Diagram of HA-tagged regions of CTCF. The central zinc fingers are indicated with numbers designating individual zinc fingers. (**B–H**) HA-CTCF proteins were transfected into HeLa cells for 48 hours followed by HSV-1 infection, and then assayed for ICP4-associated HSV-1 replication compartments. **(B)** Full-length HA-CTCF including the 1st base pair (bp) to 2184 bp of CTCF DNA sequence. **(C)** CTCF N-terminus including the 1st bp to 771 bp of CTCF DNA sequence. **(D)** CTCF N-terminus plus the DNA binding portion of the zinc finger region (Zf1-9) including the 1st bp to 1575 bp. **(E)** CTCF Zinc finger domain including 802 bp to 1731 bp. **(F)** CTCF Zinc finger plus C-terminal region including 745 bp to 2184 bp. **(G)** CTCF C-terminus region from 1593 bp to 2184 bp. **(H)** HA-CTCF lacking the Zinc fingers (Zf 1-7, 802 bp to 1380 bp).

To determine the mechanism by which CTCF is recruited to the viral replication compartment, we have defined the regions of CTCF that are necessary for its localization into HSV-1 replication compartments. The CTCF molecule was divided into several sections and HA-tags were added, generating HA-tagged the full-length CTCF, CTCF N-terminus, N-terminus plus the zinc finger region, C-terminus, C-terminus plus the zinc finger region, N- plus the C- terminus, and zinc finger region (Fig. 2A). These plasmid constructs were transfected into HeLa cells which were easier to transfect than human primary fibroblast BJ cells, followed by HSV-1 infection. As shown in Fig. 2B, the full-length HA-tagged CTCF could be readily detected in HSV-1 replication compartments using HA antibody. We found that when the zinc finger is present, either alone or in combination with the N- or the C- terminus (Fig. 2B,D,E,F), the fusion proteins colocalized with HSV-1 replication compartments, while the N- or C-terminus alone (Fig. 2C,G) showed no recruitment, even though the N- and the C-terminus were capable of nuclear localization. When the 1–7 zinc finger domain was removed from CTCF^3^, recruitment was essentially abolished as well (Fig. 2H). These data suggest that the zinc finger region is necessary for targeting CTCF to the HSV-1 replication compartments, and DNA binding by CTCF is primarily responsible for the recruitment.

### ChIP-seq revealed extensive binding of CTCF to the HSV-1 genome during lytic infection

**Figure 3:**
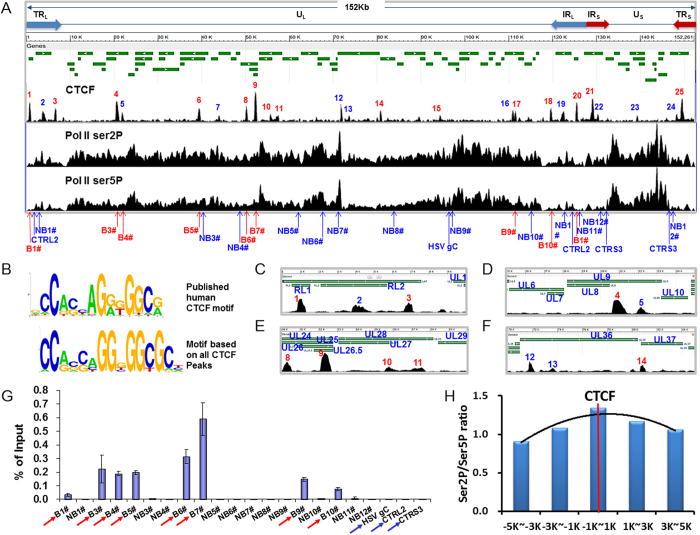
ChIP-seq analyses define multiple CTCF, pol II ser2 and pol II ser5 binding sites in the HSV-1 genome. ChIP-seq experiments using CTCF antibody was done in HSV-1 infected BJ cells at 5 MOI and 6 hpi. **(A)** Upper panel: HSV-1 genome and transcript map. Second panel: ChIP-seq of CTCF mapped onto the HSV-1 genome. A total of 25 significant CTCF binding peaks were detected. Peak number 18, 19, 20, 21 and 22 belong to internal repeat region, as a result, there are total 20 unique peaks. Third panel: ChIP seq of RNA pol II ser2P on the HSV-1 genome. Fourth panel: ChIP seq of RNA pol II ser5P on the HSV-1 genome. (**B**) CTCF motif published in ref. 11 (top) and motif based on all CTCF peaks on the HSV-1 genome (bottom). (**C**) Enlargement of 1-10k region containing peak #1 to peak #3 at gene RL1 and RL2. (**D**) Enlargement of 15–25 K region showing peaks #4 and #5. (**E**) Enlargement of 50–60k region showing peaks #8-#11. (**F**) Enlargement of 70–85 K region displaying peaks #12-#14. (**G**) 8 regions (red, arrowed) were tested to repeat the ChIP experiment followed by qPCR to validate the CTCF ChIP-seq signal. 14 randomly non-binding areas were chosen as a control. (**H**) Ratio of RNA Pol II ser2P to RNA Pol II ser5P binding signals near CTCF binding sites, p value < 2e-16.

It is not clear whether the immunofluorescent signals of CTCF colocalization with replication compartments represent its direct binding to the HSV-1 genome. To obtain direct evidence and a comprehensive picture of CTCF interaction with the lytic HSV-1 genome, we did ChIP-seq analysis of HSV-1 infected cells at 6 hpi and detected a total of 25 CTCF binding peaks on the HSV-1 genome after subtracting the control IgG reads from CTCF reads (Fig. 3A). The distribution of these peaks (Supplementary Table S2) does not show a bias towards any specific regions of viral genes as binding occurs between viral genes, at gene promoters, at 3′ end of genes or in gene bodies. For example, both the ICP0 (RL2) and ICP34.5 (RL1) genes interacted with CTCF at gene bodies (Fig. 3C). UL8, UL26 and UL36 (Fig. 3D–F) interacted with CTCF at gene promoters, while UL25 (UL24, UL26 and UL26.5), UL33 (UL34 and UL35), UL36 and UL37 bound CTCF at the 3′ end (Fig. 3E,F). The locations of several CTCF binding sites, for example, peak number 3, 9 and 14 (Fig. 3A), separate neighboring genes.

CTCF is known to interact with the host genome through a consensus sequence^11^, and it is unknown whether CTCF interacts with the HSV-1 genome through a similar mechanism. Using BioProspector software^43^, we found that the CTCF binding consensus from these peaks are highly similar to the known CTCF binding motif (Fig. 3B). Among the 25 peaks, 15 are motif positive (Fig. 3A red numbered peaks) and 10 are motif negative (Fig. 3A blue numbered peaks). The average signal intensity of motif positive peaks is higher than motif negative peaks.

We performed ChIP-qPCR to validate the ChIP-seq results by testing 8 of the CTCF peaks and 14 randomly chosen non-binding regions after HSV-1 infection for 6 hours (Fig. 3G). With the exception of #B1 primer, which showed detectable but low binding, all remaining regions encompassing CTCF peaks displayed strong CTCF enrichment. At the same time, none of the non-binding regions showed significant CTCF enrichment (Fig. 3G). Intriguingly, our ChIP-seq analysis failed to detect several previously observed CTCF binding sites during latency or post reactivation^20^,^21^,^22^. We therefore also tested 3 of these sites, CTRL2, CTa’m and CTRS3, reported as binding sites of CTCF during latency or reactivation (negative control HSV gC and DNA pol, Fig. 3G and Supplementary Figure S3) using ChIP-qPCR^21^,^22^, and found that none of these sites interacted significantly with CTCF at 2, 4 and 6 hpi.

To determine whether CTCF binding to the HSV-1 genome is dynamic during lytic infection, we also did ChIP-qPCR to measure CTCF binding at different time points after the infection, and found that there is significant occupancy of CTCF on the tested sites among 2, 4 and 6 hpi samples (Supplementary Figure S3). Since CTCF binds to HSV-1 genome as early as 2 hpi when there is no replication occurring, this result indicates that CTCF may play a role in HSV-1 lytic transcription. Taken together, these analyses suggest that CTCF interacts with the lytic HSV-1 genome when HSV-1 genomes are actively being transcribed, and that these CTCF sites may be different than those observed during latent infection or in the early stages of reactivation.

### CTCF is necessary for HSV-1 growth

**Figure 4:**
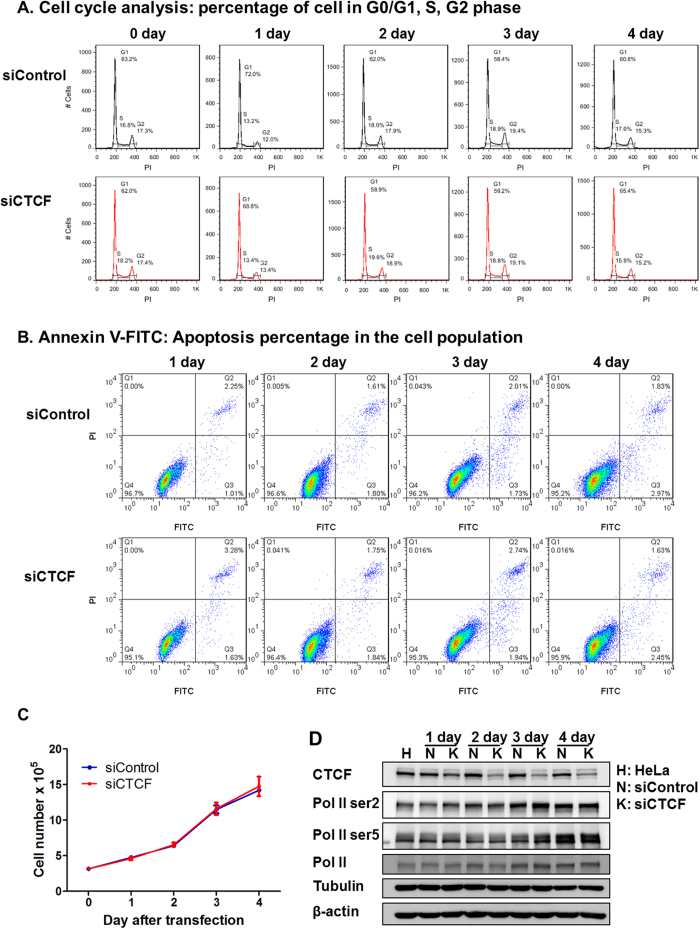
Cell cycle and apoptosis assay of the CTCF knockdown cells. HeLa cells were transfected with control siRNA or siRNA targeting CTCF for 1-4 days. HeLa cells were collected for Western blot, cell cycle and apoptosis assay. (**A**) Propidium iodide was used to stain the DNA and cell cycle was analyzed with FACS. (**B**) FITC-Annexin V was used to stain cell membrane and apoptosis of cells detected with FACS. (**C**) Cell number was detected using Countstar Automated cell Counter. (**D**) Total RNA pol II, RNA pol II ser2P and RNA pol II ser5P expression before and after CTCF knockdown were detected using Western blot. Tublin and β-actin were used as loading control. Original immunoblotting results are shown in Supplementary Figure 4.

To determine the function of CTCF in HSV-1 lytic infection, we investigated HSV-1 growth after CTCF knockdown in HeLa cells. We first analyzed the effect of the knockdown on cell proliferation, cell cycle distribution and apoptotic profile to determine the influence of CTCF knockdown on the host cell fitness. The effects of CTCF knockdown using siRNA are shown in Fig. 4D. We found that the knockdown did not affect cell cycle distribution significantly in the first 4 days, a time frame where infection experiment were to be carried out (Fig. 4A). Apoptosis and cell number in the knockdown and control cells remained similar during the course of the experiment (Fig. 4B,C), consistent with previous reports^44^,^45^. Thus, CTCF knockdown with siRNA has little effect on cell viability and cell cycle distribution in HeLa cells.

**Figure 5:**
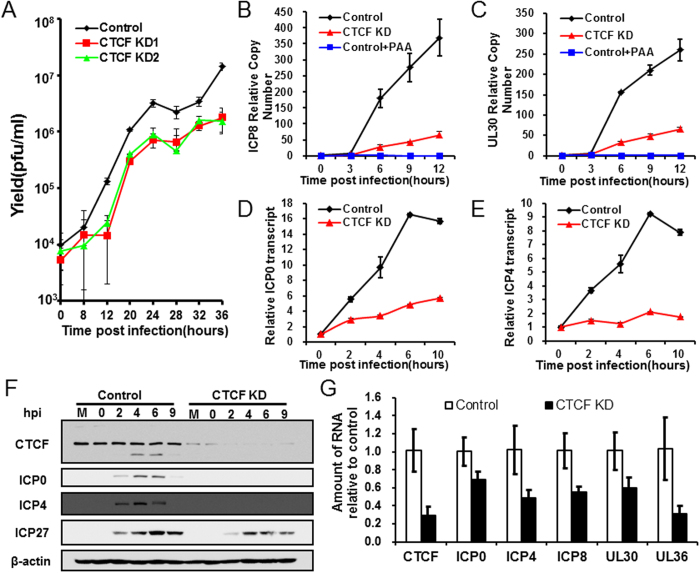
Effects of CTCF knockdown on HSV-1 proteins, transcripts, genome copy number, and virus yield. HeLa cells were transfected with control siRNA or siRNA targeting CTCF, and 3 days later, cells were infected with HSV-1. (**A)** Cells were infected with HSV-1 17+ at an MOI of 0.1 and harvested at various times post infection for determination of viral yield by plaque assay on Vero cells. (**B–E**) Cells were infected with HSV-1 17+ at an MOI of 5. Shown is DNA or mRNA fold-change over Control at 0hpi. **(B** and **C)** ICP8 and UL30 genome copy number were detected at 0, 3, 6, 9 and 12 hpi and normalized to a cellular control 18S rRNA gene. **(D** and **E)** ICP0 and ICP4 transcripts were detected at 0, 2, 4, 6 and 10 hpi and normalized to a cellular control 18S rRNA. ΔΔCt method was used to analyze qPCR data. Error bars represent standard deviation. Data are representative of 3 independent experiments. **(F)** HSV-1 IE genes ICP0, ICP4 and ICP27 were detected by W.B to show the effect of CTCF knockdown on HSV-1 protein levels. Original immunoblotting results are shown in Supplementary Figure 4. **(G)** Control cells and cells with CTCF knockdown were infected with HSV-1 strain17+ at an MOI of 5 and harvested at 6 hpi. CTCF, ICP0, ICP4, ICP8, UL30and UL36 genes transcription level were detected and normalized to a cellular control 18S rRNA. ΔΔCt method was used to analyze qPCR data. Error bars represent standard deviation. Data are representative of 3 independent experiments.

We next measured the effect of CTCF knockdown on viral growth. HeLa cells were subjected to HSV-1 infection at a multiplicity of infection (MOI) of 0.1 for 0–36 hours according to previous studies^46^,^47^, after CTCF knockdown when CTCF levels showed significant drop, and viral yield was measured at certain time points within 36 hpi. We found that starting from 12 hpi, viral titer from cells depleted of CTCF was consistently lower than from control cells, and by 36hpi, the viral titer in knockdown cells was about 10-fold lower than that in control cells (Fig. 5A). This result suggests that CTCF is important for viral growth.

The viral genome copy number was subsequently determined by qPCR using primers directed against ICP8 (Fig. 5B) and UL30 (Fig. 5C) genes. Phosphonoacetic acid (PAA) was used to inhibit HSV-1 replication as a control and indeed there was no increase for the gene copy number after adding PAA. Very little difference was detected between HSV-1 grown in the presence or reduced level of CTCF during the first 3 hours, as there is little viral replication during this time. At 6 to 9 hours, there was a significant drop of viral genome copy number in the knockdown cells. These results, together with experiments presented in Figs 4 and 5 suggest that the inhibitory effect of CTCF knockdown on viral growth is direct, and it is not due to indirect effects from cellular health.

### CTCF promotes HSV-1 lytic transcription

The reduction of viral genome copy number in CTCF knockdown cells suggests that CTCF may play a direct role on viral gene expression. As cellular RNA Pol II is needed for viral transcription, we first monitored the effect of CTCF knockdown on cellular RNA Pol II level by Western blot and found that within the duration of our experiment, RNA Pol II level was not affected (Fig. 4D). To determine how gene expression is affected, we analyzed the effect of CTCF knockdown on the transcription of two important IE genes, ICP0 and ICP4. As shown in Fig. 5D,E, a time course of ICP0 and ICP4 at 0, 2, 4, 6 and 10 hpi showed a strong reduction after CTCF knockdown, suggesting CTCF plays a positive role in immediate early gene expression. To determine whether CTCF knockdown also led to a reduction in the level of viral proteins, we did Western blot analyses of viral genes ICP0, ICP4 and ICP27 at 0 to 9 hpi, and detected significant reduction in the levels of ICP0 and ICP4, with ICP27 showing a moderate reduction (Fig. 5F). The levels of ICP27 only showed moderate reductions by the knockdown, probably because these IE genes are also subject to negative regulation by ICP4 and virion host shut off (vhs) protein^48^,^49^, which were also reduced by the knockdown (Fig. 5F,G).

We further examined several viral genes representing each kinetic class (immediate early, early and late) including ICP0, ICP4, ICP8, UL30 and UL36 at 6 hours post infection. We found that CTCF knockdown negatively affected the RNA levels of genes in all three classes (Fig. 5G). This result suggests that CTCF knockdown directly affect HSV-1 on the transcription level.

### CTCF knockdown led to increased H3K27me3, H3K9me3 binding and reduced RNA Pol II recruitment to HSV-1 genes

**Figure 6:**
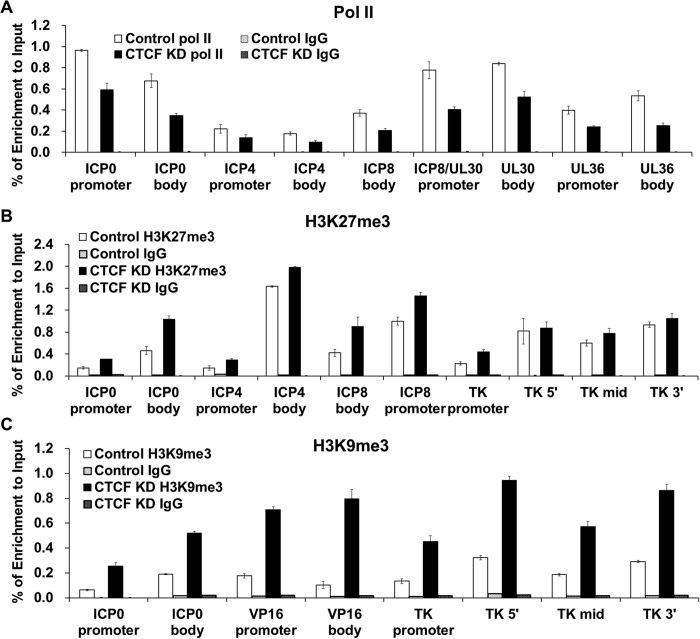
CTCF supports HSV-1 transcription by facilitating RNA pol II recruitment and preventing repressive chromatin mark H3K27me3 and H3K9me3 binding on HSV-1 genes. HeLa cells were transfected with control siRNA or siRNA targeting CTCF for 3 days. Then cells were infected with HSV-1 at 5 MOI for 2 hours (H3K27me3) or 6 hours (pol II). (**A)** ChIP using RNA pol II antibody to detect overall level of RNA polymerase II on viral genes in control and CTCF knockdown cells. Primers were designed on ICP0, ICP4, ICP8, UL30 and UL36 genes body and promoter. **(B)** ChIP using H3K27me3 antibody in Control and CTCF knockdown cells. Primers were designed at both promoter and gene bodies of ICP0, ICP4, ICP8 and TK. **(C)** ChIP using H3K9me3 antibody in Control and CTCF knockdown cells. Primers were designed at both promoter and gene bodies of ICP0, VP16 and TK genes.

To directly test the role of CTCF in viral transcription, we measured the effects of CTCF knockdown on the occupancy of RNA polymerase II (RNA pol II) on the HSV-1 genomes by ChIP-qPCR, and found that CTCF knockdown reduced the recruitment of RNA pol II to viral genes, including ICP0, ICP4, ICP8, UL30 and UL36 (Fig. 6A). The effects on RNA pol II binding to gene promoter vs. gene body for these genes appear to be similar, suggesting that the effect of CTCF knockdown is likely to be global and not restricted to specific genes or specific regions of viral genes. Since the knockdown had no effect on the level of cellular RNA Pol II (Fig. 4D), we conclude that the reduction of RNA Pol II binding to viral genome was due to the recruitment, and not the available amount of cellular RNA Pol II.

To determine if CTCF also restricted the assembly of non-permissive chromatin on the viral genome, we assayed the effect of CTCF depletion on the accumulation of the repressive histone mark H3K27me3 and heterochromatin mark H3K9me3 on HSV-1 DNA during viral infection. While there was no binding at 6hpi, we found that H3K27me3 and H3K9me3 interacted with all of the tested viral genes at 2 hpi (Fig. 6B,C), consistent with previous reports that H3K27me3 and H3K9me3 interacted with HSV-1 genes^47^,^50^. When CTCF was knocked down, the binding of H3K27me3 and H3K9me3 increased in all of the tested genes including both gene bodies and promoters (Fig. 6B,C). Taken together, these results demonstrated that CTCF played an important role in maintaining permissive chromatin structure and facilitating RNA Pol II transcription.

### CTCF promotes viral gene transcription by facilitating the elongating form of RNA Pol II binding to viral genes

**Figure 7:**
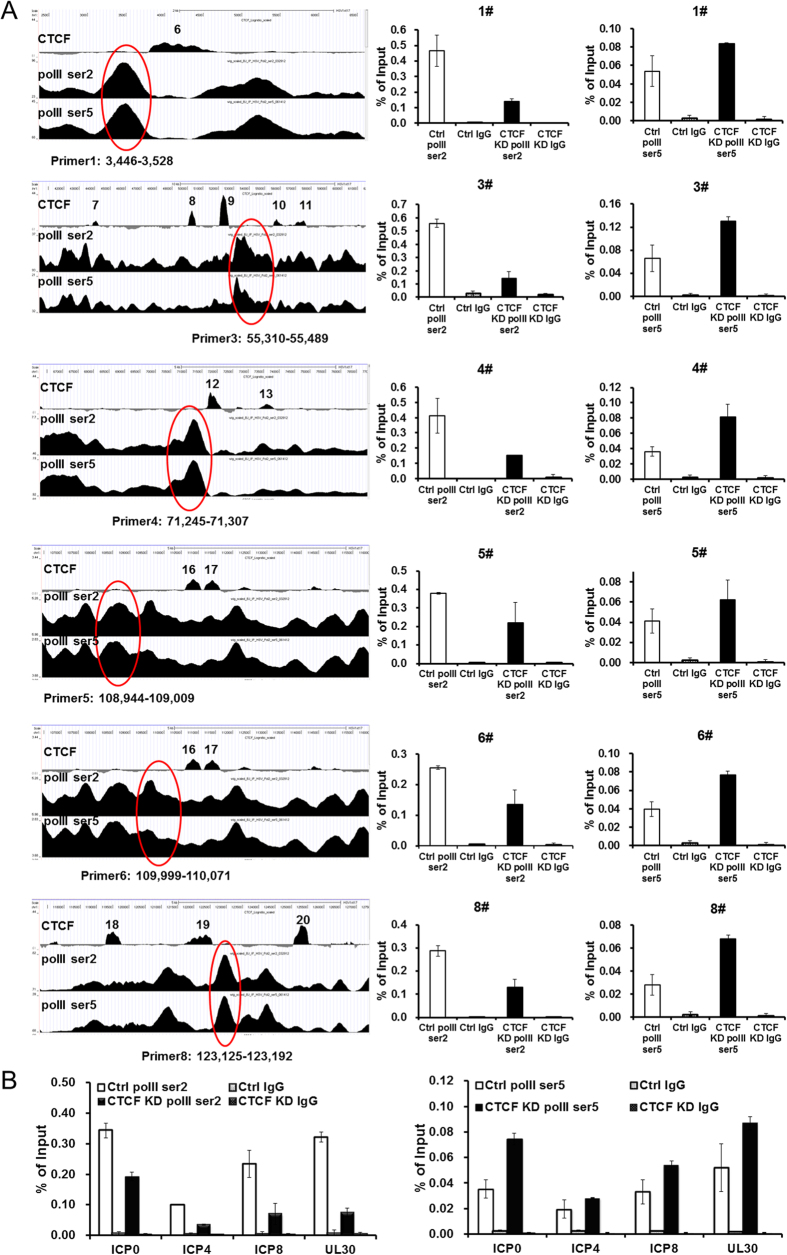
CTCF affects Pol II ser2P and pol II ser5P binding HSV-1 genes. HeLa cells were transfected with control siRNA or siRNA targeting CTCF and 3 days later, cells were infected with HSV-1 at 5 MOI for 6 hours and then were fixed for ChIP experiments. **(A)** 6 of Pol II ser2P and pol II ser5P binding peaks were chosen to perform the ChIP-qPCR. **(B)** Pol II ser2P and pol II ser5P binding peaks around ICP0, ICP4, ICP8 and UL30 were chosen to perform the ChIP-qPCR.

RNA Pol II large subunit has a C-terminal repeat domain (CTD) where serine 2 and serine 5 within these repeats could be phosphorylated by cellular CDK9 and CDK7, respectively^51^. Molecular and genome wide analyses suggested that the Ser2P modified form represents the actively transcribing, elongating RNA Pol II, while the Ser5P form represents the pausing of Pol II^52^,^53^. To determine how these modified forms of RNA Pol II interact with the lytic viral genome, we did ChIP-seq analysis of Ser2P and Ser5P form of RNA Pol II at 6 hpi, and found extensive interaction of both forms of RNA Pol II binding to the viral genome (Fig. 3A). To determine how CTCF binding relates to RNA Pol II binding to the viral genome, we compared the distribution of both phosphorylated forms of Pol II around CTCF binding sites, and found that the Ser2P form is more enriched near CTCF sites relative to the Ser5P form of Pol II (Fig. 3H), suggesting that CTCF may promote HSV-1 transcription by facilitating the elongation of RNA Pol II. To test this, we analyzed the effects of CTCF knockdown on the binding of modified forms of RNA Pol II to viral genes. We chose 6 regions where high levels of RNA Pol II binding locate beside CTCF binding sites detected by ChIP-seq and examined the effects of CTCF knockdown on the RNA Pol II forms. We found that in all 6 regions, the Ser2P form of RNA Pol II reduced after CTCF knockdown, while the Ser5P form of Pol II increased (Fig. 7A). We also examined ICP0, ICP4, ICP8 and UL30, and again the knockdown reduced the binding of Ser2P form of Pol II and increased the binding of Ser5P form of Pol II (Fig. 7B). As CTCF knockdown had no effect on cellular levels of Ser2P and Ser5P modified forms of RNA Pol II (Fig. 4D), the effects of the knockdown on modified Pol II binding to HSV-1 genes are not due to the availability of these proteins. Taken together, these results strongly suggest that CTCF facilitates the binding of the elongating form of RNA Pol II and may promote RNA Pol II elongation in the lytic HSV-1 genome.

## Discussion

Here we demonstrated that CTCF interacts with the lytic HSV-1 genome and promotes viral transcription by preventing the assembly of silenced chromatin marked by H3K27me3 and heterochromatin marked by H3K9me3 and increasing the recruitment of RNA Pol II, esp. the elongating form of RNA Pol II, to viral genes. In CTCF knockdown cells, viral gene transcription from all kinetic classes were compromised, viral replication and mature virus count were reduced.

CTCF is an essential nuclear protein, its maternal knockout in mice results in embryonic lethality by blastocyst stage, suggesting its crucial role in early developmental processes^54^. In cultured cells, the effects of CTCF knockdown vary among different cell lines and cell types. Cells isolated form CTCF conditional knockout mice, human T cells and breast cancer cell lines are sensitive to CTCF depletion^45^,^55^, resulting in an apoptosis-prone state and sensitivity to stress, while siRNA knockdown CTCF in HeLa and 293T cells are not affected^55^,^56^. One possible explanation is that compared with CTCF-null cell lines there is a small portion of CTCF remnant in siRNA knockdown cells, which help some specific type cells to survive. For this reason, we have chosen HeLa cells to do further functional analysis of CTCF knockdown on HSV-1 lytic infection. Indeed, in control experiment, we observed little or no effects on cell cycle distribution, apoptotic profile, results similar to what have been reported by others^44^,^45^,^57^ and cellular protein levels (including Ser2P and Ser5P forms of RNA Pol II) remains unchanged within the duration of our experiment of 2–4 days post knockdown (Fig. 4). Thus, the presented results are the direct effects of CTCF knockdown instead of indirect effects from alteration of cell fitness on viral lytic infection.

CTCF is known to regulate several other DNA viruses and HSV-1 itself during latency. However, the function of CTCF during HSV-1 lytic infection seems to be distinct. In EBV latency CTCF acts as a chromatin boundary to promote restricted latency and may contribute to higher order chromatin structure organization^58^,^59^. In KSHV, CTCF and cohesin initially facilitate KSHV gene transcription but inhibit lytic gene transcription at a later time, suggesting their complex roles in KSHV lytic infection^16^. In HCMV, CTCF binds to the first intron of major IE gene and functions as repressor of major IE gene expression and HCMV particle production^17^. This diversity partly reflects the multiple activities of CTCF and partially reflects the highly evolved viral strategies viruses use to take advantage of host factors. It should be noted that in HSV-1 genomes, CTCF binding sites during lytic infection differ from those during latency and reactivation (Fig. 3A,G)^21^,^22^, which further suggest complex and dynamic roles of CTCF in the life cycle of DNA viruses.

Repressive histone marks H3K9me3 and H3K27me3 can be detected on nucleosomes assembled on the viral genome at early times post infection^47^,^60^. To overcome the host silencing response, virion protein VP16 recruits host proteins, including HCF1, Oct1 and LSD1 to remove the repressive nucleosomes^61^. Transcripts of the immediate early genes are produced, including ICP4, ICP0 and ICP27, after disruption or removal of nucleosomes^62^,^63^. ICP4 interacts with multiple components of the pol II machinery, such as the Mediator complex, TATA box-binding protein (TBP), TFIIB and TBP-associated factor 1 to regulate viral gene expression^64^. ICP27 is reported to recruit RNAP II to viral genes promoters, although it mainly inhibits host pre-mRNA export^62^. In CTCF knockdown cells, ICP27 is only moderately affected, therefore, the reduction of RNAP II recruitment to viral genes could not be solely explained by ICP27.

In contrast, ICP22 is reported to degrade the CTD ser2P form of RNAP II^28^, leading to the proposal that viral transcription maybe less dependent on the cellular mechanism to enhance transcriptional elongation^65^. However, it is also reported that the CTD ser2P form of RNA Pol II is indeed required for HSV-1 transcription, as CDK9 deficiency impaired HSV-1 growth^30^,^66^. Here we demonstrated ser2P and ser5P forms of RNA Pol II binding to HSV-1 genome by ChIP-seq and ChIP-qPCR, and revealed that there is an enrichment of the Ser2P form over the ser5P form of RNA Pol II near CTCF binding sites in the lytic HSV-1 genome (Fig. 3H). Subsequent CTCF knockdown experiment resulted in an increase of ser5P form and a reduction of Ser2P form of RNA Pol II on viral genes, while the overall RNA Pol II level on these genes were also reduced. This experiment strongly supports a role for CTCF in promoting RNA Pol II elongation on viral genes.

CTCF acts at multiple levels to control gene expression: from acting as transcription repressor, activator, insulator, to organizer of chromatin loops and topological domains^3^. CTCF has been seen to interact with transcription enhancers^67^, upstream of gene promoters^68^,^69^, and is hypothesized to facilitate enhancer-promoter interactions, a proposal that contradicts with its insulator activity per se^3^. Direct molecular studies on the relationship between CTCF and RNA Pol II suggested that CTCF interacts with RNA Pol II through the C terminus^8^. It also interacts with p-TEF to facilitate Pol II elongation^70^. However, a genome wide study suggest that one group of CTCF binding site in gene intron exon junctions may lead to RNA Pol II pausing^7^. How CTCF facilitates Pol II elongation on the lytic HSV-1 genome is not known at this point, and it is an important topic for further analysis. Several possible explanations could be envisioned. First, CTCF may directly recruit RNA Pol II and/or p-TEF to the viral genome. The increased local concentration of Pol II, esp. the CTD ser2P-modified form of Pol II, could promote viral gene transcription, while the presence of CTCF could prevent silenced chromatin from interacting with the viral genome. This possibility is supported by the RNA Pol II ChIP experiment in Fig. 6A. The second possibility, is that CTCF may act through its 25 binding sites to organize the viral genome into a defined 3D structure, in a way much like the active transcription hub seen in the β-globin locus^71^, to facilitate viral transcription and to effectively fend off host defenses by keeping silenced chromatin away. The increased repressive nucleosomes mark H3K27me3, H3K9me3 and reduced RNA Pol II binding to the viral genome after CTCF knockdown is consistent with this possibility (Fig. 6). A third possibility is that the reduction of CTCF levels down regulated viral factors such as ICP0 and ICP4 as a result of increased silencing, and indirectly reduced Pol II recruitment to the viral genome^62^,^64^,^72^.

## Materials and Methods

The methods were carried out in accordance with the approved guidelines.

### Cells and virus

BJ, HeLa, 293T and Vero cells were obtained from American Type Culture Collection. Cells were grown in Dulbecco’s modified Eagle’s medium (DMEM; Gibco) supplemented with 10% fetal bovine serum (FBS), penicillin (100 U/ml), and streptomycin (100 μg/ml) in a humidified 5% CO2 atmosphere at 37 °C. Virus was grown and titrated on Vero cells. Viral infections were done at indicated MOI. Briefly, cultured cells were replaced with serum free DMEM, followed by adding the virus and incubating for 1 hour with occasional rotation to get an even spread, then the culture medium was replaced by regular DMEM with 10% FBS and 1% antibiotics. All experiments were carried out in accordance with the approved guidelines of ethics committee of Kunming Institute of Zoology, and all experimental protocol were approved by ethics committee of Kunming Institute of Zoology, Chinese Academy of Sciences.

### Antibodies

CTCF polyclonal antibodies (A002202) were bought from Abcam (ab70303) or made by GL Biochem (Shanghai), CTCF monoclonal antibodies were from Millipore. Antibodies against RNA Pol II, RNA Pol II Ser2P, RNA Pol II Ser5P, H3K27me3, H3K9me3, HA, ICP8, ICP27 were from Abcam. Monoclonal antibody against ICP4 is a gift from Gerd Maul’s laboratory at the Wistar Institute^73^,^74^. Alexa Fluor 594 Goat Anti-Mouse IgG (H+L) Antibody and Alexa Fluor 488 Goat Anti-Rabbit IgG (H+L) Antibody were from Life Technologies.

### Immunofluorescence

BJ and HeLa cells were seeded on glass coverslips in 24-well plates one day before infection and used for infections at an MOI of 5. At 5 or 6 hpi, cells were fixing with 4% paraformaldehyde at 4 °C for 60 min and extracted with 0.2% Triton X-100 in PBS for 10 min. Nuclei were visualized by staining with Hoechst33342. Images were acquired using Nikon 80i or Nikon N-SIM.

Images were taken with different channels for different samples. Images were merged and processed by Image J^75^. To quantify the signal of a certain signal in a certain region a polygon selection tool was used to determine the regions. Then we applied the “measure RGB” tool to the selected regions under the Plugins tab in Image J menu. Then we got the mean signal strength of each channel in the region. The collected signals were analyzed with two tailed t-test tool in excel. Error bars represent standard errors (s.e.m.).

### CTCF deletion mutant cloning

CTCF deletion mutants were made by PCR using the primers in Supplementary Table S3. PCR products were subsequently cloned into HA-tagged vector (pCMV4-3HA).

### siRNA mediated knockdown of CTCF

siRNAs targeting GTAGAAGTCAGCAAATTAA of CTCF were transfected to HeLa cells using Lipofectamine 2000 (Life Technologies, 11668019) according to the manufacturer’s instructions. HSV-1 infection was done after siRNA transfection for 48–72 hours. Western blot detection of CTCF protein levels was done 2–4 days after lentivirus transfection.

### Isolation host and viral genomic DNA and RNA for qPCR and qRT-PCR analyses

HeLa cells were infected with HSV-1 at an MOI of 5 and harvested at various time points. To purify genomic DNA, Genomic DNA purification kit (DP304-03, Tiangen) was used. For RNA purification, TRIzol (Ambion, 15596-018) was used. 1 μg RNA was reverse transcribed using Prime ScriptRT Reagent Kit with gDNA Eraser (TaKaRa, DRR047A) and stored in −20 degree. Real time PCR was run in triplicate with 50ng cDNA or 50ng genomic DNA using FastStart Universal SYBR Green Master (Roche, 04913914001) and ABI7900HT. Sequences of primers used are provided in the Supplementary Table S1. Viral DNA or RNA levels at each time point were quantified relative to the 0 hpi samples by the ΔCt method. To determine the relative DNA or RNA content at various times, average Ct valued for ICP0, ICP4, ICP8 and UL30 genes were subtracted by the average Ct walues for 18 s. The calibrator value (HSV sample 0 hpi) was subtracted by the 18 s Ct value. To obtain the ΔΔCt value, the Ct value was subtracted by the Ct value of the input time point. ΔΔCt = (Ct_test_ − Ct_reference_) − (Ct_0 hpi sample_ − Ct_0 hpi 18s_). The fold enrichment value is 2^−ΔΔCt^.

### Cell cycle assay

Cells were collected and fixed with 75% v/v ethanol at 4 °C overnight. The cells were re-suspended in PBS containing propidium iodide (PI, 20 μg/ml) and RNase A (10 μg/ml) at 37 °C in the dark for 30 minutes. Cell cycle distribution was analyzed by flow cytometry (Becton Dickinson LSR Fortessa, USA). Data from 10,000 cells per sample were collected.

### Apoptosis assay

Cells were collected, washed and stained with Annexin-V FITC kit according to the manufacturer’s protocol. Briefly, cells were collected and washed twice with 1X binding buffer and incubated in 100 μl labeling solution containing 1 μl annexin-V FITC conjugate and 10 μl PI in the dark for 15 min at room temperature. The fluorescence of the samples was analyzed by flow cytometry (Becton Dickinson LSR Fortessa, USA). Data from 10,000 cells per sample were collected.

### Western blot

BJ cells were either mock-infected or infected with HSV-1 at an MOI of 5. At 0, 2, 4, 6, 9 hpi, cells were harvested after two washes in ice-cold PBS, and whole-cell lysates were prepared. Protein concentration was estimated by Lowry assay (Bio-Rad), and 30 μg of protein was loaded per well. Western blotting was carried out by standard protocols. The following antibodies were used: ICP0 monoclonal (Maul laboratory, 1:1,000); ICP4 monoclonal (Maul laboratory, 1:500); ICP27 monoclonal (Maul Laboratory, 1:1,000); CTCF polyclonal (GL Shanghai A002202, 1:1,000) and β-actin monoclonal (Abcam ab8224, 1:3,000). Detection was done using Super Signal West Pico Chemiluminescent Substrate (Thermo, OH192607). Original immunoblotting results are shown in Supplementary Figure 4.

### Chromatin Immunoprecipitation

ChIP assays were carried out according to the protocol from Chromatin Immunoprecipitation Assay Kit (Millipore) with minor modification. BJ, HeLa cells were infected with HSV-1 at an MOI of 5. At 4hpi or 6hpi cells were fixed with formaldehyde (Sigma, final concentration 1% v/v). Then Glycine (125 mM) was added to stop the reaction. Cells were washed 3 times with ice-cold PBS then scraped from culture dishes into a microfuge tubes. Cells were collected by centrifugation at 5,000 × g at 4 °C for 10 minutes. The cells were lysed by Lysis Buffer with protease inhibitors and sonicated to yield DNA fragments of between 200 bp and 500 bp in length. The samples were clarified by centrifugation at 13,000 × g at 4 °Cfor 15 min. The supernatant was diluted 10-fold in IP Dilution Buffer with protease inhibitors. An aliquot (1/20) of each chromatin supernatant was reserved as the input sample. Dynabeads Protein G from INVITROGEN with a magnetic stand was used for immunoprecipitation. The chromatin supernatant was incubated with 5 μg antibody specific for pol II or 5 μg antibody specific for CTCF overnight at 4 °C with rotation. An aliquot was incubated with IgG (Abcam, ab2410) as a control to determine background binding. The beads were washed for 5 min at 4 °C with rotation, twice with Low-salt Buffer, once with High-salt Buffer, once with LiCl Buffer, twice with TE Buffer. Immunocomplexes were eluted by adding 210 μl of Elution Buffer incubating for 15 min at 65 °C. Spin the beads at 13,000 rpm for 1 min and take 200 μl of eluted solution and transfer to a new tube. Crosslinks were reversed by incubation for 7 hours at 65 °C with a final concentration of 200 mM NaCl. The samples were then treated with RNase A and digested with proteinase K. DNA was purified by QIA quick PCR Purification Kit (QIAGEN, Cat. No 28104), and used as a template for real-time PCR or sequencing (Sequencing was done at the Wistar Genomic Core facility).

### Data analysis

CTCF ChIP-seq data was aligned to HSV-1 genome (GenBank: JN555585.1) with Bowtie^76^ and using BED tools to convert the sam format file to Bed Graph format and uploading to IGV with default argument. Then we normalized it by dividing total aligned reads in a given bin (around 1000 bp) and multiplying it by million. To determine CTCF binding motif, we first extract the sequences of the peaks with 100 bp window. The BioProspector was applied to the extracted sequences. BioProspector parameters were -n 80 -a 1 -h^43^. 25 unique peaks were classified into two types: motif positive peaks (1, 3, 4, 6, 8, 9, 10, 11, 14, 15, 17, 18, 20, 21 and 25) and motif negative peak (2, 5, 7, 12, 13, 16, 19, 22, 23 and 24).

## Additional Information

**How to cite this article**: Lang, F. *et al*. CTCF interacts with the lytic HSV-1 genome to promote viral transcription. *Sci. Rep.* **7**, 39861; doi: 10.1038/srep39861 (2017).

**Publisher's note:** Springer Nature remains neutral with regard to jurisdictional claims in published maps and institutional affiliations.

## Supplementary Material

Supplemental Materials
