# Author Correction: CTCF interacts with the lytic HSV-1 genome to promote viral transcription

**DOI:** 10.1038/s41598-021-84469-2

**Published:** 2021-02-25

**Authors:** Fengchao Lang, Xin Li, Olga Vladimirova, Benxia Hu, Guijun Chen, Yu Xiao, Vikrant Singh, Danfeng Lu, Lihong Li, Hongbo Han, J. M. A. S. P. Wickramasinghe, Sheryl T. Smith, Chunfu Zheng, Qihan Li, Paul M. Lieberman, Nigel W. Fraser, Jumin Zhou

**Affiliations:** 1 Key Laboratory of Animal Models and Human Disease Mechanisms of the Chinese Academy of Sciences & Yunnan Province, Kunming Institute of Zoology, Kunming, Kunming, 650223 China; 2 Kunming College of Life Science, University of Chinese Academy of Sciences, Kunming, Beijing, 100101 China; 3 Gene Expression and Regulation Program, The Wistar Institute, Philadelphia, PA 19104 USA; 4 Biology & Chemistry Engineering College, Panzhihua University, Panzhihua, 617000 Sichuan China; 5 Department of Biology, Arcadia University, Glenside, PA 19038 USA; 6 Institutes of Biology and Medical Sciences, Soochow University, Suzhou, 215123 China; 7 Department of Microbiology, Immunology and Infectious Diseases, University of Calgary, Calgary, AB T2N 4N1 Canada; 8 Department of Viral Immunology, Institute of Medical Biology, Chinese Academy of Medicine Science, Peking Union Medical College, Kunming, Kunming, 650118 China; 9 Department of Microbiology, Perelman School of Medicine, University of Pennsylvania, Philadelphia, PA 19104 USA

Correction to: *Scientific Reports* 10.1038/srep39861, published online 03 January 2017

The Acknowledgements section in this Article is incomplete.

"We thank Nikon Instrument (Shanghai) CO., LTD. & Chansn Instrument (China) LTD. for technical support with super resolution microscopy. We also thank Dr. Matthew D. Weitzman for sharing reagents. This work was supported by grants from Chinese Academy of Sciences (KSCXZ-EW-BR-6), Startup fund from Kunming Institute of Zoology, Chinese Academy of Sciences (Y102421081), Grants from Yunnan Provincial Government (2013FA051; 2011HA005), National Science Foundation of China (NSFC 81471966) to JZ, a NSFC grant to YX (31200964), a Common Project of Panzhihua, Science and Technology Bureau of China (2012CY-S-22(9)) to HH and a Visiting professorship for senior international scientist from CAS to NWF (2012T1S0001)."

should read:

"We thank Nikon Instrument (Shanghai) CO., LTD. & Chansn Instrument (China) LTD. for technical support with super resolution microscopy. We also thank Dr. Matthew D. Weitzman for sharing reagents. This work was supported by grants from National Science Foundation of China (NSFC U1602226, 81672040, 81471966), Chinese Academy of Sciences (KSCXZ-EW-BR-6), Startup fund from Kunming Institute of Zoology, Chinese Academy of Sciences (Y102421081), Grants from Yunnan Provincial Government (2013FA051; 2011HA005) to JZ, a NSFC grant to YX (31200964), a Common Project of Panzhihua, Science and Technology Bureau of China (2012CY-S-22(9)) to HH and a Visiting professorship for senior international scientist from CAS to NWF (2012T1S0001)."

