# Supplementary material for: CTCF interacts with the lytic HSV-1 genome to promote viral transcription
Source: Sci Rep. 2017 Jan 3;7:39861. doi: 10.1038/srep39861 (PMC5206630; doi:10.1038/srep39861)
Supplement: Supplemental Materials [file srep39861-s1.pdf]

Title:

CTCF interacts with the lytic HSV-1 genome to promote viral transcription

Authors/Affiliations:

Fengchao Lang[1,2,*], Xin Li[1,2,*], Olga Vladimirova[3], Benxia Hu[1,2], Guijun Chen[1], Yu Xiao[1], Vikrant Singh[3], Danfeng Lu[1,2], Lihong Li[1], Hongbo Han[4], J.M.A.S.P Wickramasinghe[3], Sheryl T. Smith[5], Chunfu Zheng[6,7], Qihan Li[8], Paul Lieberman[3], Nigel W. Fraser[9] and Jumin Zhou[1]

[1] Key Laboratory of Animal Models and Human Disease Mechanisms of the Chinese Academy of Sciences & Yunnan Province, Kunming Institute of Zoology, Kunming, Kunming 650223, China;

[2] Kunming College of Life Science, University of Chinese Academy of Sciences, Kunming, Beijing 100101, China;

[3] Gene Expression and Regulation Program, The Wistar Institute, Philadelphia, PA 19104, USA;

[4] Biology & Chemistry Engineering College, Panzhihua University, Panzhihua, Sichuan 617000, China;

[5] Department of Biology, Arcadia University, Glenside, PA 19038, USA;

[6] Institutes of Biology and Medical Sciences, Soochow University, Suzhou, 215123, China;

[7] Department of Microbiology, Immunology and Infectious Diseases, University

of Calgary, Calgary, AB T2N 4N1, Canada;

[8] Department of Viral Immunology, Institute of Medical Biology, Chinese Academy of Medicine Science, Peking Union Medical College, Kunming, Kunming 650118, China;

[9] Department of Microbiology, Perelman School of Medicine, University of Pennsylvania, Philadelphia, PA 19104, USA.

* These authors contributed equally to the manuscript.

Correspondence and requests for materials should be addressed to J.Z. (email: zhoujm@mail.kiz.ac.cn).

Supplemental Figures and Tables

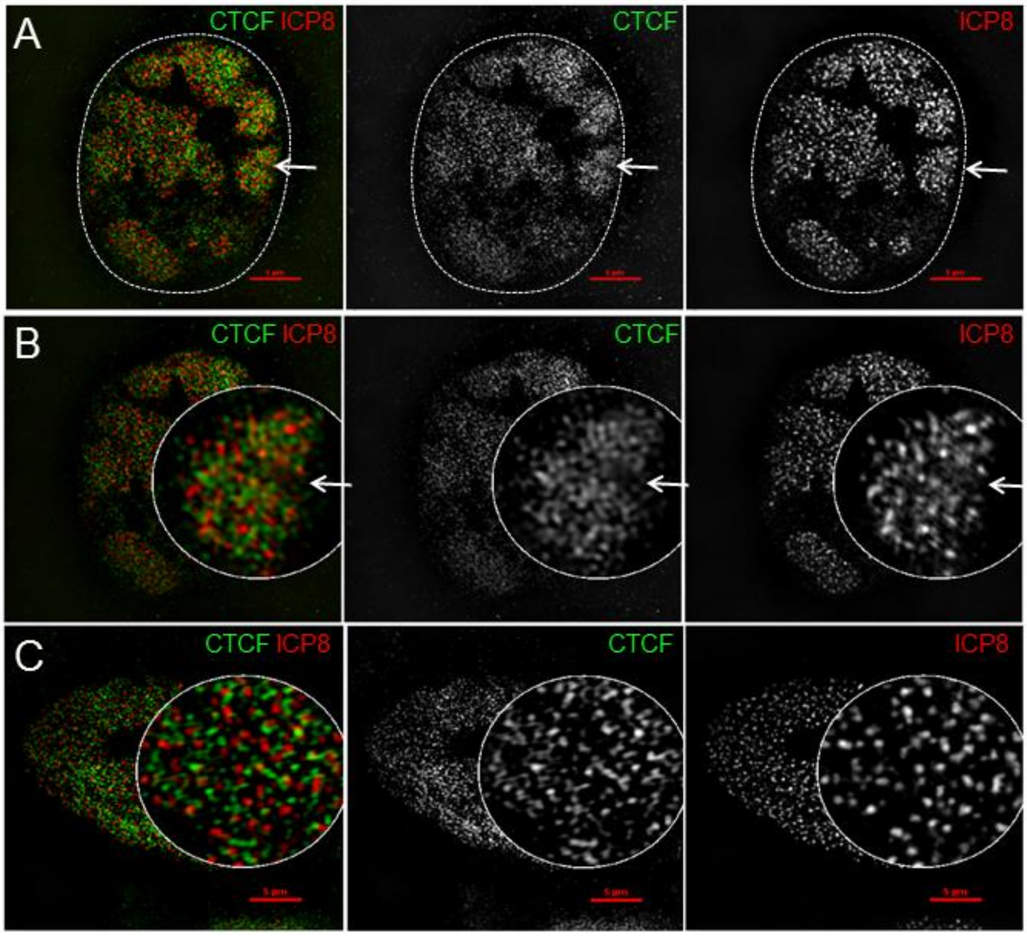

Figure S1. Distribution of CTCF and ICP8 in HSV-1 replication compartments.

(A) Double staining of CTCF (green) and ICP8 (red). Images were obtained through Nikon N-SIM super resolution microscopy. Arrow points to a HSV-1 replication center. **(B)** An enlargement of the replication compartment from A (see arrow in A). **(C)** Another view showing the location of CTCF and ICP8.

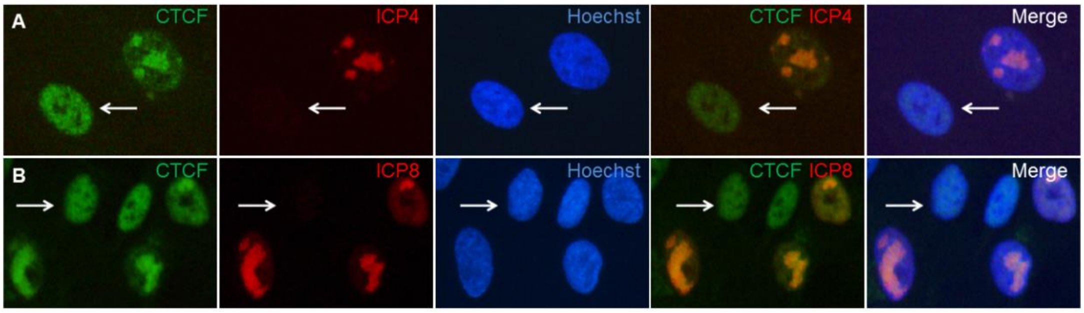

Figure S2. Colocalization of CTCF and ICP4 or ICP8 in HSV-1 replication compartments under the traditional fluorescent microscopy.

(A) Double staining of CTCF (green) and ICP4 (red). White arrow points to uninfected cells. **(B)** Double staining of CTCF (green) and ICP8 (red). Images were obtained through Nikon 80i microscopy. White arrow points to uninfected cells.

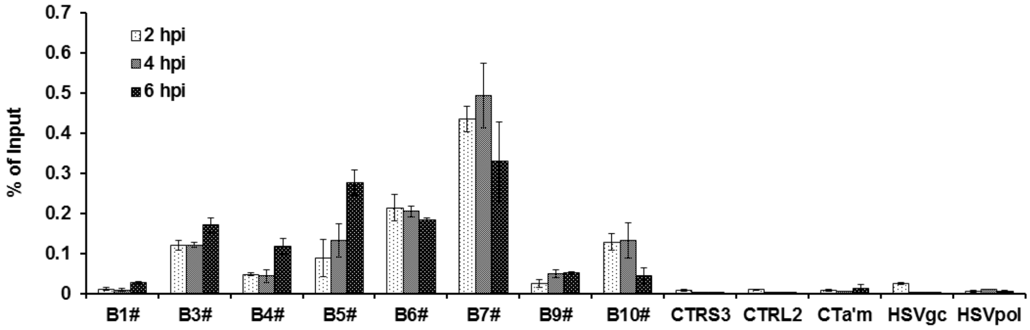

Figure S3. CTCF binding sites in the HSV-1 genome at 2, 4 and 6 hours post infection.

Human primary epithelial fibroblast BJ cells were infected with HSV-1 17+ at an MOI of 5 for 2, 4, 6 hours and then were collected for ChIP experiments. 8 of CTCF binding peaks were chosen to repeat the ChIP experiment followed by qPCR to validate the CTCF ChIP-seq signal. 5 non-binding areas were chosen as a control.

Figure 4D

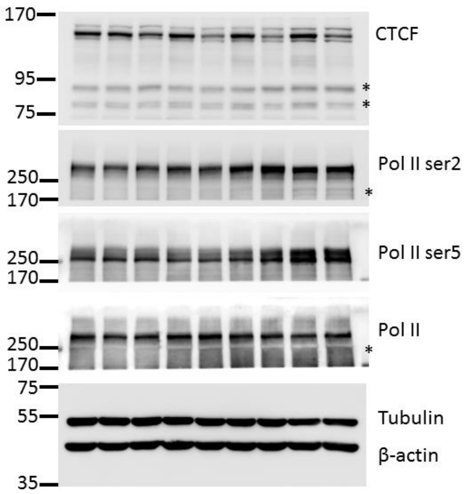

Figure 5F

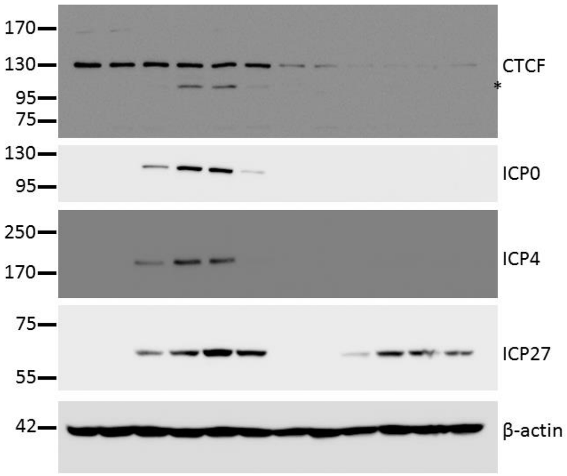

Figure S4. Original immunoblotting results for figures 4D and 5F.

***Non-specific bands**

Table S1. Sequences of primers used for Real-time PCR

HSV CTCF peak B1# F	TCTAACGTTACACCCGAGGC
HSV CTCF peak B1# R	GTATATATGCGCGGCTCCTG
HSV CTCF peak B3# F	TACGTTACAGTGGCCCAGTT
HSV CTCF peak B3# R	GATGACGTGCTGGACCTACT
HSV CTCF peak B4# F	GACTGGTACACGGACACCAT
HSV CTCF peak B4# R	CGATCCCCTGCACTTTGATG
HSV CTCF peak B5# F	GATCAAACAGCGCCTCCTG
HSV CTCF peak B5# R	CTCCTCCTGCATCTCCAGC
HSV CTCF peak B6# F	CTGGAACTCATCAACCGCAC
HSV CTCF peak B6# R	AAGCCCCTGTTCGTATTGGA
HSV CTCF peak B7# F	GGGACCAACGAGACCATCA
HSV CTCF peak B7# R	ACCGGCGTGTACATTCCATA
HSV CTCF peak B9# F	TCCTGTTGATCGATACGGGG
HSV CTCF peak B9# R	ATCACAAACACTGGCAGCAG
HSV CTCF peak B10# F	TTAGACACCTGCTTCTCCCC
HSV CTCF peak B10# R	GAAAAGAACGGGCTGGTGTG
HSV-1 CTRL2 F	CCGCGGCTCTGTGGTTA
HSV-1 CTRL2 R	GGATGCGTGGGAGTGGG
HSV-1 CTa’m F	GCTGCCACAGGTGAAACC
HSV-1 CTa’m R	TGTAGCAGGAGCGGTGTG
HSV-1 CTRS3 F	ATCGCATCGGAAAGGGACACG
HSV-1 CTRS3 R	CCAAGGTGCTTACCCGTGCAAA
HSV-1 gC F	CCTTGCCGTGGTCCTGTGGA
HSV-1 gC R	GGTGGTGTTGTTCTTGGGTTTG
HSV-1 DNApol F	AGAGGGACATCCAGGACTTTGT
HSV-1 DNApol R	CAGGCGCTTGTTGGTGTAC
18s F	GTAACCCGTTGAACCCCATT
18s R	CCATCCAATCGGTAGTAGCG
CTCF F	GACCCCACCCTTCTTCAGATG
CTCF R	CCACAGCAGCCTCTGCTTCT

ICP0 F	CTGCGCTGCGACACCTT
ICP0 R	CAATTGCATCCAGGTTTTCATG
ICP4 F	GCCGGGGCGCTGCTTGTTCTCC
ICP4 R	CGTCCGCCGTCGCAGCCGTATC
ICP8 F	GAGACCGGGGTTGGGGAATGAATC
ICP8 R	CCCGGGGGGTTGTCGGTGAAGG
UL30 F	TGTTTCGCGTGTGGGACATA
UL30 R	TTGTCCTTCAGGACGGCTTC
UL36 F	GTAACAGGCGCGGATCAGTA
UL36 R	AAGAGGTGACGCGCTTACAA
ICP0 promoter F	CGCTTCCCGGTATGGTAATTAGAAAC
ICP0 promoter R	CGTGTGTTCCGCCAAAAAAGCAATTAGC
ICP4 promoter F	ATCGCATCGGAAAGGGACACG
ICP4 promoter R	CCAAGGTGCTTACCCGTGCAAA
ICP8/30 promoter F	GATCCGCCAGACAAACAAGG
ICP8/30 promoter R	AAAGAGGGGAATGTGGGGAG
UL36 promoter F	GGGTCTTAAGCACCTACGGA
UL36 promoter R	CCAACCACCCACCAAATAGC
HSV ser2/5 primer 1# F	CCGTGCATGAAAACCTGGAT
HSV ser2/5 primer 1# R	TCACGCCCACTATCAGGTAC
HSV ser2/5 primer 3# F	GTGGCCTTGAACTTGTACGG
HSV ser2/5 primer 3# R	GACATCAAGGCGGAGAACAC
HSV ser2/5 primer 4# F	AATTGTAAGCGCGTCACCTC
HSV ser2/5 primer 4# R	ACCAACGAACCATTCAGTGC
HSV ser2/5 primer 5# F	CGGATCATCTCATATTGTTCCTC
HSV ser2/5 primer 5# R	GCTTCTCTTCTCGGGGCTA
HSV ser2/5 primer 6# F	TACGTGGAGCGCCGTATT
HSV ser2/5 primer 6# R	GCGATCGTGGGTTATGAACT
HSV ser2/5 primer 8# F	AGCCCCGTCTCGAACAGT
HSV ser2/5 primer 8# R	ACCACCATGACGACGACTC

Table S2. Identified CTCF peaks and consensus location on HSV-1 genome.

The CTCF wig peak file was loaded to IGV to visualize the CTCF bindings on HSV-1 genome. A window of 200 bp was sliding through the HSV-1 genome to localize the CTCF peaks manually and the actual size of the window was adjusted according to the width of a certain CTCF peak. The CTCF peaks were classified into two types, i.e. high peaks or low peak. The sequences of CTCF peaks were extracted accordingly. The extracted sequences were applied to BioProspector (30). The sequences of CTCF peaks and CTCF motifs were listed.

**Peak number**	**Sequence**	**Motifs exist or not (15bp)**
**1** (85bp)	GTCCGGGTGCGCCACCTGGTGGTCTGGGCCTCGGCC GCCCGCCTGGCGCGCCGCGGCTCGTGGGCCCGCGA GCGGGCCGACCGGG(1062-1146)	CCACCAGGT GGCGCA
**2** (77bp)	CGGACAGCGCCCCCCTCGGCGCCCATCGGGCCACAC GGCAGCAGTAACACCAACACCACCACCAACAGCAGC GGCGG(4003-4079)	None
**3** (81bp)	CTATTGGGGAGAAGCAGGTGTCTAACCTACCTGGAAA CGCGGCGTCTTTGTTGAACGACACCGGGGCGCCCTC GACGAGTG(6880-6960)	ACACCGGG GCGCCCT
**4** (183bp)	GGATGATGTCACGTTGGGGTTGGCGCTGGATGAGTG CGTGCGCAAACAGCGCCCCCACGCGGGCACGCGTAG CTTGAAGCGCGCGCCCGCAGCCATCTGCACGCCAAA CAGTTCGCCCCAGATTATCTTGAACAGCGCCACCGCG TGGTCCGTCTCGCTAACGGACCCGCGCGGGGGACAG CC(21027-21118)	1.CCACGCG GTGGCGCT 2.CCGCGTG GGGGCGCT
**5** (92bp)	CCGTCCATGTAAATCAGTAGCTCCCCCTTGCGGAGGG TGCGCACCCGTCCCAGGGACTGGTACACGGACACCA TGTCCGGTCCGTAGTTCAT(22056-22147)	None

**6**<br>(420bp)	GCCCACCAGGGGGTAGGGAACGTTGGTGGCGGCGTAGATGCGCTTCTCCAGGGCCTCCAGAAAGACCAGCTTCTCGCCGATGGACACCAGATCCGCGCGCACGCGCGTCGTCTGGGGGGCGCTCTCGAGCTCGTCCAGCGTCTGCCGGTTCAGGTCGAGCTGCTCCTCCTGCATCTCCAGCAGGTGGCGGCCCACGTCGTCCAGACTTCGCACGGCCTTGCCCATCACGAGCGCCGTGACCAGGTTGGCCCCGTTCAGGACCATCTCGCCGTACGTCACCGGCACGTCGGCTTCGGTGTCCTCCACTTTCAGGAAGGACTGCAGGAGGCGCTGTTTGATCGGGGCGGTGGTGACGAGCACCCCGTCGACCGGCCGCCCGCGCGTGTCGGCATGCGTCAGACGGGGCACGGCCACGGAGGG(39342-39761)	CCAGCAGGTGGCGGC
**7**<br>(120bp)	TAGCAACAAGGCCGTGGACGGCACGTCGCTCGAAAACACGCTTGGGGCGCCCTCCGTCGGCCCGGCGGCGATTTGCTGCTGTGTGTTGTCCGTATCCACCAGCAACACAGACATGACCTC(44041-44160)	None
**8**<br>(92bp)	GGGTCGACGCGGCGGGTGGTGGATATGTCATCGGGGGCCCGCCAGGCGGCGCTGGTGCGCCTCACCGCCCTGGAACTCATCAACCGCACCCG(50254-50345)	CCGCCAGGCGGCGCT
**9**<br>(191bp)	ACCCGTACTACCCCGGGGAGGCTCGAGGCGGGCAGCGCGGGGTCGACTCTCGGCGCGCGGCCCGCCAGTCTCCCGGGACCAACGAGACCATCACGGCGCTGATGGGGGCGGTGACGTCTCTGCAGCAGGAACTGGCACACATGCGGGCTCGGACCAGCGCCCCCTATGGGATGTACACGCCGGTGGCACAC(52152-52342)	CCCATAGGGGGCGCT
**10**<br>(120bp)	CGCCACCAGGACCCCCAGCGTCAACCCCAAGAGCGCCCATACGACGAACCACCGGCGCCCCCGCGCGGGGGCGCCCTGGCGCATGGCGGGACTACGGGGGCCCGTCGTGCCCCCCGTCAG(55712-55831)	CCGCGCGGGGGCGCC(with human CTCF concensus as background)

**11** (720bp)	CTGTTCGGGGCTGACGAGGCGCCGCAAGAGCGGCGT CGTCAGGTGGTGGTCGTAGCACGCGCGGATGAGCGC CTCGATCTGATCGTCGGGTGACGTGGCCTGACCGCC GATTATTAGGGCGTCCACCATATCCAGCGCCGCCAGG TGGCTCCCGAACGCGCGATCGAAATGCTCCGCCCGC CGCCCGAACAGCGCCAGTTCCACGGCCACCGCGGCG GTCTCCTGCTGCAACTCGCGCCGCGCCAGCGCGGTC AGGTTGCTGGCAAACGCGTCCATGGTGGTCTGGCCG GCGCGGTCGCCGGACGCGAGCCAGAATCGCAATTCG CTGATGGCGTACAGGCCGGGCGTGGTGGCCTGAAAC ACGTCGTGCGCCTCCAGCAGGGCGTCGGCCTCCTTG CGGACCGAGTCGTTCTCGGGCGACGGGTGGGGCTG CCCGTCGCCCCCCGCGGTCCGGGCCAGCGCATGGT CCAACACGGAGAGCGCCCGCGCGCGGTCGGCGTCC GACAGCCCGGCGGCGTGGGGCAGGTACCGCCGCAG CTCGTTGGCGTCCAGCCGCACCTGCGCCTGCTGGGT GACGTGGTTACAGATACGGTCCGCCAGGCGGCGGGC GATCGTCGCCCCCTGGTTCGCCGTCACACACAGTTCC TCGAAACAGACCGCGCAGGGGTGGGACGGGTCGCTA AGCTCCGGGGGGACGATAAGGCCCGACCCCACCGCC CC(56910-57629)	CCACCTGGC GGCGCT(with human CTCF concensus as background)
**12** (110bp)	GTGGCCACCGGCTGCCGTGTAGTGGGGCGGCGGGA AACCGGGCCTCCGGGCGCAACACCGCCCTCCAGCGT CAAGTATGTGGGGGGCGGGCCTGACGTCGGGGGCG GGGC(71869-71978)	None
**13** (270bp)	ACAGCGTGAGACACAGCTCCAGCTCCGACTCGCGGG AAAAGGCCGTGGTGTTGCGGAGCGCCACGACGACGG GCGCGCCCAGGAGCACTGCCGCCAGCACCAGGTCCA TGGCCGTAACGCGCGCCGCGGGGGTGCGGTGGGTG GCGGCGGCCGGCACGGCGACGTGCTGGCCCGTGGG CCGGTAGAGGGCGTTGGGGGGAGCGGGGGGTGACG CCTCGCGCCCCCCCGAGGGGCTCAGCGTCTGCCCAG ATTCCAGACGCGCGGTCAGAA(73415-73684)	None
**14** (160bp)	GTCCGTGGGTGGCGCGACGGTGTCCTCCGGGATCAT CTCGTCGTCCTCGACGGGGGTGTTGGAATGAGGCGC CCCCTCGCGGTCCGCCTGGCGTGGGCCGTGCCCATA GGCCTCCGGCTTCTGTGCGTCCATGGGCATAGGCGC GGGGAGACTGTTTCCG(80737-80896)	CCGCGAGG GGGCGCC(with human CTCF concensus as background)

**15** (100bp)	GGGGCCTTGCATGCCTGTCCGATGCCGTCGTCGACCACGCGGGTCACTCCCACGACCAAGCGGGGGCGCTCGGGGGGCGAGGATGCGCGCGGACACGG(94234-94333)	CCAAGCGGGGGCGCT(with human CTCF concensus as background)
**16** (92bp)	GGCTCACGGATCGCATCCGCGCCCAGGGCCCCGTGATGCCCCCTGGCGGCCTGGATGCCGGGGGGCAGATGTACGTGAATCGCAACGAGATA(110802-110893)	None
**17** (92bp)	TCGTCCACGGTTCTCTAACGATGCGGGGGGTGGCACGGGTCATCCAGCAGGCGGTGCTGTTGGACCGAGATTTTGTGGAGGCCATCGGGAGC(111366-111457)	CCAGCAGGCGGTGCT
**18** (90bp)	CCCAAACGACAGGGGGCGCCCCAGAGGCTAAGGTCGGCCACGCCACTCGCGGGTGGGCTCGTGTTACAGCACACCAGCCCGTTCTTTTCC(119505-119594)	ACGACAGGGGGCGCC
**19** (130bp)	TGTTGGTGTTACTGCTGCCGTGTGGCCCGATGGGCGCCGAGGGGGGCGCTGTCCGAGCCGCGGCCGGCTGGGGGGCTGCGTGAGACGCCCCGCCCGTCACGGGGGGCGCGGCGGCGCCTCTGCGTGGGGG(122317-122446)	None
**20** (91bp)	GGCGCGCCAGGCGGGCGGCCGAGGCCCAGACCACCAGGTGGCGCACCCGGACGTGGGGCGAGAAGCGCACCCGCGCGGGGGTCGCGGGGGT(125261-125351)	CCACCAGGTGGCGCA
**21** (92bp)	CGGCGTCCGGTGCGCTGGCCGCCGCCGCCAGCAGGGGGCGCAGGCTCTGGTTGTCAAACAGCAGGTCCGCGGCGGCGGCGGCCGCGGAGCTC(129001-129092)	CCAGCAGGGGGCGCA
**22** (148bp)	CCGAGGCCTCGAACCGGCGTCGCGCCTCCTCCGCCTCGGGCGCCCCCCAGAGGCCCGGGCGGCTGTCGCCCAGGCCGCCGTACAGCACCCGCCCCGGGGGCGGGGGCCCGGCGCCGGGCCACGGCTCCCCGCTGACGTACCCGTCGCG(130066-130213)	None
**23** (180bp)	GAGCACCGAGCCAAGGGCTCCTGTAAGTACGCCCTCCCGCTGCGCATCCCCCCGTCAGCCTGCCTCTCCCCCCAGGCCTACCAGCAGGGGGTGACGGTGGACAGCATCGGGATGCTGCCCCGCTTCATCCCCGAGAACCAGCGCACCGTCGCCGTATACAGCTTGAAGATCGCCGGGTGG(139041-139220)	None
**24** (90bp)	GCGGGTGCTGTACGGCGGCCTGGGCGACAGCCGCCCGGGCCTCTGGGGGGCGCCCGAGGCGGAGGAGGCGCGACGCCGGTTCGAGGCCTC(148040-148129)	None
**25** (92bp)	ACCTGCTGTTTGACAACCAGAGCCTGCGCCCCCTGCTGGCGGCGGCGGCCAGCGCACCGGACGCCGCCGACGCGCTGGCGGCCGCCGCCGCC(149131-149222)	CCAGCAGGGGGCGCA

Table S3. Sequences of primers used for CTCF deletion mutants

CTCF Full	CTCF N-F	CCCAAGCTTCGGAAGGTGATGCAGTCGAAGC
	CTCF Full-R	CGCGGATCCTCACCGGTCCATCATGCTGA
CTCF NR	CTCF N-F	CCCAAGCTTCGGAAGGTGATGCAGTCGAAGC
	CTCF N-R	CGCGGATCCAATTTTTGTTGGCTTTGGAG
CTCF NZn	CTCF N-F	CCCAAGCTTCGGAAGGTGATGCAGTCGAAGC
	CTCF N+Zn-R	CGCGGATCCGCAGGCGTAAGGCTTCTCCC
CTCF Zn	CTCF Zn-1F	CCCAAGCTTGTAAAGAAGACATTCCAG
	CTCF Zn-1R	GCTCTAGATCAGCCAGCACAATTATCAGC
CTCF ZnC	CTCF C+Zn-F	CCCAAGCTTCGAAAAGCCACACTGATGAGAG
	CTCF Full-R	CGCGGATCCTCACCGGTCCATCATGCTGA
CTCF CR	CTCF C-1F	CCCAAGCTTCGAGCCACTGCGATAAGACCTT
	CTCF Full-R	CGCGGATCCTCACCGGTCCATCATGCTGA
CTCF ʌZn1-7	CTCF ʌZn1-7	CCCAAGCTTGCAGGGGAAATGGAAGGT
	CTCF ʌZn1-7	CGCGGATCCCTGGAATGTCTTCTTTAC
	CTCF ʌZn1-7	CGCGGATCCATTGAGCAAGGCAAGAAA
	CTCF ʌZn1-7	GCTCTAGA AAGGCTCCGCCATCACCG
